# Escape from homeostasis: spinal microcircuits and progression of amyotrophic lateral sclerosis

**DOI:** 10.1152/jn.00331.2017

**Published:** 2018-01-31

**Authors:** Robert M. Brownstone, Camille Lancelin

**Affiliations:** Sobell Department of Motor Neuroscience and Movement Disorders, Institute of Neurology, University College London, London, United Kingdom

**Keywords:** excitotoxicity, α-motoneurons, γ-motoneurons, muscle spindles, proprioceptive afferents, Renshaw cells

## Abstract

In amyotrophic lateral sclerosis (ALS), loss of motoneuron function leads to weakness and, ultimately, respiratory failure and death. Regardless of the initial pathogenic factors, motoneuron loss follows a specific pattern: the largest α-motoneurons die before smaller α-motoneurons, and γ-motoneurons are spared. In this article, we examine how homeostatic responses to this orderly progression could lead to local microcircuit dysfunction that in turn propagates motoneuron dysfunction and death. We first review motoneuron diversity and the principle of α-γ coactivation and then discuss two specific spinal motoneuron microcircuits: those involving proprioceptive afferents and those involving Renshaw cells. Next, we propose that the overall homeostatic response of the nervous system is aimed at maintaining force output. Thus motoneuron degeneration would lead to an increase in inputs to motoneurons, and, because of the pattern of neuronal degeneration, would result in an imbalance in local microcircuit activity that would overwhelm initial homeostatic responses. We suggest that this activity would ultimately lead to excitotoxicity of motoneurons, which would hasten the progression of disease. Finally, we propose that should this be the case, new therapies targeted toward microcircuit dysfunction could slow the course of ALS.

## INTRODUCTION

Many diverse provinces of the central nervous system are involved in the production of movement, and, through their interconnections, the coordination of activity of circuits in these regions leads to organized behavior. Microcircuits within and between many regions of the cerebral cortex, basal ganglia, cerebellum, brain stem, and spinal cord each play a role in movement, whether as selection circuits, command neurons, organization circuits, or the final common path leading to muscle contraction. These circuits are remarkably adaptive: movement is well controlled in a multitude of environmental conditions. To do so, multiple modalities of sensory input are involved in the moment-to-moment adjustments of motor output to ensure appropriate coordination and function of these disparate motor circuits.

There are homeostatic processes in neurons and circuits that ensure that the output of neurons, in the form of trains of action potentials, is maintained within a specific range (Turrigiano and Nelson 2004) needed for the behavior. This homeostatic regulation is necessary to maintain activity throughout the life cycle of an organism, for example, in relation to the short timescale of protein turnover (Marder and Goaillard 2006; O’Leary et al. 2014). In the vertebrate motor system, for example, it is necessary to maintain muscle force production in the range necessary for movement (e.g., consider body weight support). Thus homeostatic processes in neurons (motoneurons) and circuits underlie homeostatic processes of the organism (movement) (see Fig. 2*A*).

Homeostatic mechanisms also play important roles in maintaining movement following damage to the nervous system. For example, after spinal cord injury, spinal circuits can regain activity needed for locomotor function (Bui et al. 2016; Martinez et al. 2011). Similarly, homeostasis is also seen in neurodegenerative diseases, in which symptoms do not become apparent until a significant proportion of neurons dies. For example, Parkinson’s disease is asymptomatic until an estimated 30% of nigral dopaminergic neurons die (Fearnley and Lees 1991; Greffard et al. 2006; Ma et al. 1997), and amyotrophic lateral sclerosis (ALS) remains asymptomatic until at least 30% of vulnerable motoneurons (MNs) degenerate (Lalancette-Hebert et al. 2016; Zang et al. 2005). In some pools, up to 70% of motor units may have degenerated by the time of symptom onset (Hegedus et al. 2007). Thus circuit homeostasis plays an important role in maintaining quality of life in the face of neurological disease or injury.

Conversely, there are situations in which circuit homeostasis may be maladaptive. For example, following spinal cord injury, adaptations including changes in MN serotonin receptors (Murray et al. 2010) and/or chloride homeostasis (Boulenguez et al. 2010) lead to spasticity, sometimes to a degree that can significantly impair quality of life (Holtz et al. 2016), and plasticity of autonomic motor systems can lead to autonomic dysreflexia (reviewed in Brown and Weaver 2012), which can be life-threatening. Understanding the mechanisms of maladaptive plasticity is thus important for the development of strategies to improve quality of life in people with neurological diseases.

In this review, we ask whether maladaptive plasticity of motor circuits can contribute to progression of neurodegenerative diseases. We focus on ALS, describing two fundamental spinal circuits involving MNs. We do not suggest that circuit dysfunction is causative of ALS, but rather propose that ALS-induced changes in these circuits disrupt normal homeostatic mechanisms and may thus accelerate the progression of MN degeneration. We present an hypothesis whereby maladaptive plasticity of MN circuits leads to excessive glutamate receptor activation, excitotoxicity, and hence further MN dysfunction and, ultimately, death. We therefore suggest that the development of strategies to target microcircuits involved in these maladaptive processes could slow the progression of disease.

## SELECTIVE VULNERABILITY OF MOTONEURONS IN ALS

ALS was described by Charcot in the 19th century (Charcot and Joffroy 1869). ALS is a fatal adult-onset neurodegenerative condition associated with progressive loss of MNs, leading to weakness and eventually death by respiratory failure (for review, see Kiernan et al. 2011). Although ALS can affect other central nervous system functions, we focus on MN degeneration.

The underlying causes of ALS are not clear and are not explored in this review. Various cellular and molecular hypotheses have been proposed to explain MN death, e.g., aggregation of toxic proteins, defects in RNA metabolism, or disrupted axonal transport (for review, see Peters et al. 2015; Taylor et al. 2016). Importantly, the death of MNs is not considered to be a cell autonomous process (Ditsworth et al. 2017).

The clinical presentations of ALS are heterogeneous. For example, initial MN loss may be in the brain stem (bulbar) or spinal cord. The time of onset varies considerably, although it is most commonly diagnosed between the 6th and 8th decades of life, and the speed of progression is quite variable (for review, see Swinnen and Robberecht 2014). Despite these differences, there are some commonalities in the pathology. For example, some MN types are vulnerable to degenerative processes, whereas others are resistant (reviewed in Nijssen et al. 2017), and the motor symptoms and signs of ALS tend to start in a certain location and spread to adjacent regions in an orderly manner (reviewed in Ravits 2014).

Regarding selective vulnerability, the pattern of MN loss is remarkably consistent regardless of the etiology of the disease. In the spinal cord, MNs of the lateral motor column (LMC) are affected to a greater extent than those of the medial motor column (MMC). Furthermore, sacral MNs that innervate external anal and urethral sphincter muscles (Onuf’s nucleus) are spared (Iwata and Hirano 1979; Mannen et al. 1977; Schrøder and Reske-Nielsen 1984). In the brain stem, ALS affects trigeminal MNs that innervate the muscles of mastication, facial MNs that supply the superficial muscles of the face, hypoglossal MNs innervating the muscles of the tongue, and ambiguus MNs supplying the muscles of the soft palate, pharynx, and larynx. In contrast, oculomotor, trochlear, and abducens MNs (II, IV, VI nuclei) innervating the extraocular muscles are spared (Iwata and Hirano 1979; Nimchinsky et al. 2000; Valdez et al. 2012), as is the parasympathetic dorsal motor nucleus of the vagus (Iwata and Hirano 1979). The reasons why some populations are spared are not clear (Hedlund et al. 2010), and although not addressed directly by our hypothesis, the circuits that we discuss, as we point out below, are different in these populations. We emphasize that our hypothesis is related to disease progression rather than causation. Of note, there is recent evidence that the factors underlying disease onset and progression in ALS are different (Ditsworth et al. 2017).

There is also selective vulnerability within motor pools, the populations of MNs that innervate a single muscle. α-MNs, those that innervate extrafusal muscle fibers responsible for force production, are vulnerable, whereas γ-MNs, those that innervate the contractile elements of muscle spindles to regulate proprioceptive feedback, are resistant to degeneration (Kawamura et al. 1981; Lalancette-Hebert et al. 2016; Mohajeri et al. 1998; Vaughan et al. 2015). Furthermore, the largest α-MNs that innervate fast-twitch muscle fibers degenerate before the smaller α-MNs that innervate slow-twitch muscle fibers (Frey et al. 2000; Pun et al. 2006). Again, the mechanisms underlying this orderly death are not clear.

## MOTONEURON TYPES

Given these differences in MN vulnerability, we next explore local microcircuits involving different types of MNs. Spinal MNs, termed the “final common path” for movement by Sherrington (1904), receive and integrate inputs from supraspinal, spinal, and sensory neurons and project axons outside the central nervous system to innervate muscles and thus effect movement. Despite this common role, they do not constitute a uniform population.

**Fig. 1.**
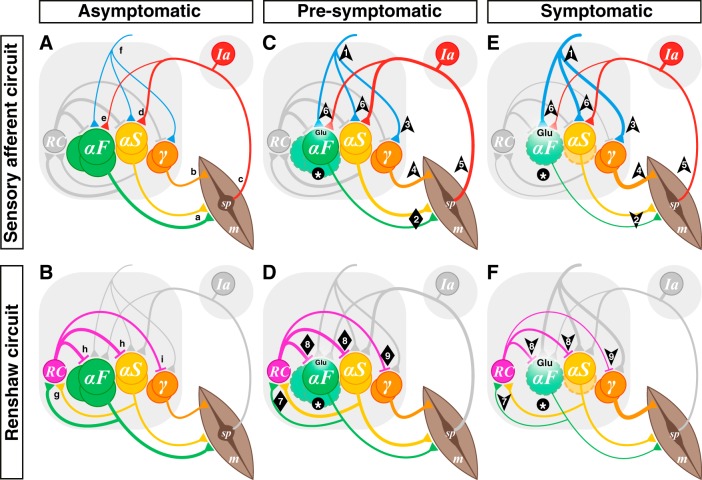
Hypothesis: α-motoneuron (α-MN) death leads to microcircuit imbalance and disease progression. Sensory afferent circuits (*A*, *C*, *E*) and Renshaw circuits (*B*, *D*, *F*) in asymptomatic (*A*, *B*), presymptomatic (*C*, *D*), and symptomatic (*E*, *F*) amyotrophic lateral sclerosis (ALS). *A* and *B*: normal spinal MN circuits. MN pools comprise multiple types: α-MNs can be defined by the extrafusal muscle fiber (m) types they innervate as either fast (αF; FF and FR types depicted together) or slow (αS) (*a*). γ-MNs innervate muscle spindles (sp; *b*), which convey length and velocity information back to α-MNs primarily via group Ia afferents (*c*), which form monosynaptic connexions with α-MNs (*d*, *e*). During most movement, α- and γ-MNs are coactivated by spinal and supraspinal neurons (*f*). α-MNs also innervate Renshaw cells (RC; *g*), which in turn inhibit both α- (*h*) and γ-MNs (*i*). *C* and *D*: in presymptomatic ALS, α-MNs (F-type) become dysfunctional and start to die (*), but γ-MNs are preserved. Homeostatic mechanisms include increased input to α-MNs from spinal and supraspinal circuits (*1*) to ensure that force production is preserved (*2*). Thus the input to the coactivated γ-MNs would also increase (*3*), leading to increased intrafusal fiber contraction (*4*) out of proportion to extrafusal fibers. This α-γ imbalance would result in an increase in spindle afferent input to α-MNs (*6*). The increasing glutamatergic (Glu) excitation from these inputs would initially maintain the homeostatic response despite a reduction of activity of fast high-force-producing muscle fibers. In addition, the loss of α-MNs (particularly type F; *) would concomitantly lead to a reduction of output from MN pools to RCs, initially compensated by increased α-MN activity (particularly type S; *7*). Thus Renshaw inhibition would at first be maintained in all MN types (*8*, *9*). Together, these processes would lead to increased glutamatergic excitation of vulnerable α-MNs and, hence, excitotoxicity. *E* and *F*: in symptomatic stages, type F α-MNs continue to die and type S α-MNs start to degenerate and ultimately die at later stages of the disease (*), but γ-MNs are completely spared. The processes that started in presymptomatic stages would continue, there would be runaway from homeostatic processes, and further excitotoxicity would lead to disease progression. It would no longer be possible to maintain muscle contraction (*2*), compounding the α-γ imbalance (*2* and *4*, *5*), and the resulting loss of input to RCs (*7*) would reduce Renshaw inhibition of α-MNs (*8*) and also diminish γ-MN inhibition (*9*), thereby contributing to increased excitation of remaining α-MNs but a further imbalance of α-γ output. Note that the thickness of each line represents the integral of synaptic transmission over the number of synaptic contacts on the target cells. In the interest of simplicity and clarity, static and dynamic γ-MNs are represented as a single population, group II sensory afferents are not shown, and MN types are represented as distinct groups, although they are intermingled in each MN pool. Arrows indicate direction of change, and diamonds indicate no net change.

It is perhaps useful to consider MN types from an evolutionary standpoint. After the evolution of contractile muscles and their innervating α-MNs (Fig. 1*A*, *c*), muscle sensory feedback evolved, with the development of muscle spindles and associated afferent fibers to relay stretch length and velocity data back to the central nervous system (for review, see Manuel and Zytnicki 2011) (Fig. 1*A*, *c*). Spindles developed contractile elements such that their tension could be regulated during muscle shortening. Early in vertebrate evolution, spindle contractile elements were innervated by the same MNs (β-MNs) that innervated extrafusal fibers (Adal and Barker 1965; Bessou et al. 1965), but in mammalian evolution, the roles divided such that many spindles became innervated by an independent class of MNs termed γ-MNs (Burke et al. 1977; Kuffler et al. 1951) (Fig. 1*A*, *b*). About 30% of most MN pools are composed of γ-MNs, which overlap in size with small α-MNs, have simpler dendritic branching patterns (Westbury 1982), and can be distinguished by diminished expression of NeuN (Friese et al. 2009; Shneider et al. 2009). The intermixture of α- and γ-MNs in each motor pool is similar from rostral to caudal pools (Burke et al. 1977).

Two main classes of γ-MNs contribute to the modulation of muscle proprioceptive feedback. Static γ-MNs innervate nuclear chain and bag_2_ fibers and regulate the stretch sensitivity of primary and secondary endings (Ia and II sensory afferents) conveying feedback related to length. Dynamic γ-MNs innervate nuclear bag_1_ fibers and regulate the dynamic sensitivity of primary endings (Ia sensory afferents) conveying information on lengthening velocity (Brown and Butler 1973; Jansen and Matthews 1962; Matthews 1962; Murphy 1982). Thus, during behavior, α-MNs produce movement and γ-MNs regulate sensory feedback from muscles.

A second type of diversification of MNs is that α-MNs themselves are not homogeneous. α-MN types can be defined by the contractile properties of the muscle fibers they innervate (Bakels and Kernell 1993; Gardiner 1993; Manuel and Heckman 2012), with each MN forming synapses with muscle fibers of similar structural and functional properties (Edström and Kugelberg 1968). Thus a single α-MN innervates multiple similar muscle fibers, which together constitute a motor unit, the functional unit of the motor system (Buchthal and Schmalbruch 1980; Liddell and Sherrington 1925). Accordingly, α-MNs can be divided into three main subtypes: slow (S), fast fatigue resistant (FR), and fast fatigable (FF) (Burke et al. 1973). S-MNs form synapses with slow-twitch fatigue-resistant type I muscle fibers, forming type S motor units; FR-MNs form synapses with fast-twitch fatigue-resistant type IIa (fast oxidative) muscle fibers, constituting FR motor units; and FF-MNs form synapses with fast-twitch (fast glycolytic) fatigable type IIb muscle fibers, forming FF motor units (Burke et al. 1973; McDonagh et al. 1980). Within a motor pool innervating a muscle with a mixture of fiber types, α-MNs of various types are intermingled with each other (Burke et al. 1977) (and with γ-MNs).

The different types of MNs have different biophysical, morphological, and molecular properties (for a detailed review, see Kanning et al. 2010; Stifani 2014). In general, type S motor units are responsible for low-amplitude forces of long duration, whereas FF motor units are responsible for high-amplitude force ballistic movements. Corresponding to these forces, S-MNs have longer postspike afterhyperpolarizations (AHPs) (Eccles et al. 1957a), fire at lower frequencies than F-MNs (Kernell 1979; Zengel et al. 1985), and can produce prolonged self-sustained tonic firing (Lee and Heckman 1998), whereas FF-MNs have shorter AHPs and fire at higher frequencies and in phasic discharge patterns (Burke 1968a). These MN properties thus correspond to the muscle fiber types they innervate (Bakels and Kernell 1993; Eccles et al. 1958; Schiaffino and Reggiani 2011).

In response to uniform input to a motor pool, there is orderly recruitment of MNs from S to FR to FF (Denny-Brown and Pennybacker 1938; Henneman 1957). This results from their electrophysiological properties: type S MNs are smaller (Cullheim et al. 1987; Ulfhake and Kellerth 1982) and have higher input resistances and lower rheobase currents (Kernell and Monster 1981; Zajac and Faden 1985; Zengel et al. 1985). Thus multiple MN types are coordinated in a specific manner to produce a given movement.

## INPUTS TO MOTONEURONS: α-γ COACTIVATION AND BALANCE

During muscle contraction, the activity in α- and γ-MNs is generally balanced to prevent spindle unloading and thus maintain spindle sensitivity. The initial evolution of β-MNs ensured coactivation of spindles and extrafusal muscle fibers. The emergence of γ-MNs provided the central nervous system with some control over proprioceptive feedback, potentially leading to a form of “active sensing.”

Nonetheless, in many behaviors, there is evidence in animals and humans that α- and γ-MNs are, at least to a degree, coactivated (for review, see Prochazka and Ellaway 2012). Many different inputs (spinal and supraspinal) lead to coactivation of α- and γ-MNs (Granit 1975; Grillner 1969; Sjöström and Zangger 1975, 1976; Vallbo et al. 1979) (Fig. 1*A*, *f*). Although there is some variability, in many rhythmic movements α- and γ (both static and dynamic)-MNs are coactivated (Ellaway et al. 2015; Murphy and Martin 1993). For example, during locomotion, α-γ coactivation has been shown in both extensor (Bessou et al. 1986) and flexor (Bessou et al. 1990) motor pools (Edgerton et al. 1976). This coactivation could promote a degree of servo-assisted muscle contraction (Hagbarth 1993; Watanabe and Hirayama 1976). In support of this, sectioning of γ-MN efferents in cats led to a reduction of α-MN activity evoked by supraspinal inputs (Severin 1966). In general, coactivation of α- and γ-MNs is necessary to prevent spindle unloading that would occur with extrafusal muscle fiber contraction alone (Murphy and Martin 1993).

It should be noted that it is not a rule that α- and γ-MNs are always coactivated; if that were the case, then there would have been no evolutionary pressure to move beyond β-MNs to the independent control afforded by γ-MNs. There are several examples of differential control of α- and γ-MNs. First, group Ia spindle afferents, which comprise the major direct proprioceptive inputs to MNs, form synapses with and thus activate only α-MNs, thus avoiding a possible positive feedback loop that would be created with γ-MN activation. Second, spinal circuits differentially activate static vs dynamic γ-MNs during locomotion, with each type serving a different purpose (Ellaway et al. 2015). Third, selective inputs to γ-MNs would allow the nervous system to independently adjust afferent sensitivity during different behaviors (Prochazka et al. 1985; Vallbo and Hulliger 1981). This has been nicely demonstrated during an attention task (Hospod et al. 2007). Thus, although α-γ coactivation is common during movement, the nervous system has the ability to tune the balanced relationship between contraction and spindle sensitivity by weighting inputs to either α- or γ-MNs.

## SENSORY FEEDBACK CIRCUITS

Muscle spindles act as stretch-sensitive mechanoreceptors to provide information about muscle length and lengthening velocity to the nervous system (Hunt 1951). These data are transmitted by groups Ia and II proprioceptive sensory afferents.

Group Ia afferents project directly to homonymous and synergist α-MNs (Brown and Fyffe 1981; Eccles 1946; Lloyd 1943a, 1943b, 1946a, 1946b) (Fig. 1*A*, *d* and *e*), but not to γ-MNs (Appelberg et al. 1983; Eccles et al. 1960; Friese et al. 2009; Shneider et al. 2009). A single Ia afferent fiber contacts virtually all α-MNs of the homonymous pool, with each MN receiving afferents from almost all spindles of the muscle it innervates (Brown and Fyffe 1978, 1981; Mendell and Henneman 1968). The strength of heteronymous connections is typically weaker than that of homonymous connections (Burke and Glenn 1996; Eccles et al. 1957b; Mendelsohn et al. 2015).

The strength of monosynaptic group Ia excitation differs on different MN types. Whereas the average number of sensory collaterals and boutons is similar on all MN types (Burke and Glenn 1996), the strength of Ia excitation varies according to MN size. That is, Ia excitation is strongest on type S MNs (Fig. 1*A*, *d*) and weakest on type FF MNs (Fig. 1*A*, *e*) (Burke 1968b; Burke and Rymer 1976; Eccles et al. 1957b; Heckman and Binder 1988).

There is evidence that group II afferents also project directly to homonymous MNs, but these connections are weak (Fyffe 1979; Hongo 1992; Kirkwood and Sears 1974). Contrary to group Ia afferents, group II afferents project to only about one-half of homonymous MNs, and the resulting excitation is equal across α-MN types (Munson et al. 1982). In addition, although γ-MNs receive few primary sensory afferent inputs (Shneider et al. 2009), about one-third of γ-MNs seem to receive monosynaptic input from group II afferents converging from a variety of muscles (Gladden et al. 1998), although there is minimal anatomical evidence of this, with few proprioceptive boutons apposing γ-MNs (Shneider et al. 2009). Thus primary and secondary spindle afferents provide direct excitation to MN pools, but with different distributions.

Not all motor pools receive direct spindle afferent input. In the spinal respiratory motor columns, for example, despite the presence of a few spindles in the diaphragm (Corda et al. 1965b), there is no monosynaptic afferent input to phrenic MNs; thus phrenic MNs do not respond to muscle stretch (Corda et al. 1965a). On the other hand, intercostal muscles have many spindles (Huber 1902) and intercostal MNs (which are MMC MNs) receive monosynaptic homonymous Ia connections (Corda et al. 1965a; Kirkwood and Sears 1982). In the brain stem, facial MNs do not have spindle afferent input (there are no spindles in facial muscles), but trigeminal MNs, innervating the muscles of mastication, do. Thus innervation of MNs by primary afferents varies across the neuraxis.

Monosynaptic connectivity between proprioceptive afferents and MNs has been less well studied in ALS-resistant motor pools. The external urethral and anal sphincters have few, if any, spindles (Chennells et al. 1960; Garry and Garven 1957; Todd 1964; Walker 1959), including in humans (Lassmann 1984a, 1984b). Although MNs in Onuf’s nucleus showed weak monosynaptic responses to dorsal root stimulation (Mackel 1979), no anatomical evidence of proprioceptive afferent input directly to these MNs has been found (e.g., Lalancette-Hebert et al. 2016). The presence of proprioceptive feedback from extraocular muscles is even less clear. Extraocular muscles of some species completely lack muscle spindles, whereas in others, their number and morphology vary considerably (for review, see Büttner-Ennever et al. 2006; Donaldson 2000; Maier et al. 1974). These muscles contain palisade endings that may function as proprioceptors (Dogiel 1906; Lienbacher and Horn 2012). However, it seems clear that there are no proprioceptive afferents from extraocular muscles that form monosynaptic connexions with extraocular MNs (Keller and Robinson 1971; reviewed in Rao and Prevosto 2013). Thus ALS-resistant MN pools get little, if any, monosynaptic proprioceptive feedback directly from the muscles they innervate.

## RENSHAW CELL CIRCUITS

α-MNs have targets in addition to muscle: they have axon collaterals that form synapses with inhibitory interneurons in the ventral horn, Renshaw cells (Alvarez et al. 1999; Lagerbäck et al. 1981; Lagerbäck and Ronnevi 1982; for review, see Alvarez and Fyffe 2007) (Fig. 1*B*, *g*). A single Renshaw cell is excited by axon collaterals from several α-MNs, which can be from different motor pools (Eccles et al. 1954, 1961b; Moore et al. 2015; Renshaw 1946).

Renshaw cells are differentially innervated by different types of α-MNs, with the relative contribution from larger MNs being greater: the ratio of type S, FR, and FF MNs to the excitation of Renshaw cells is ~1:2:4 (Cullheim and Kellerth 1978; Hultborn et al. 1988). In contrast to α-MNs, however, γ-MNs almost completely lack axon collaterals (Westbury 1982) and hence have minimal, if any, contribution to Renshaw inhibition (for review, see Windhorst 1990).

Renshaw cells have several targets (Hultborn et al. 1971; Ryall 1970; Wilson et al. 1964), but in this review we focus on their MN targets. Renshaw cells project back to and form inhibitory synapses (glycinergic and/or GABAergic) with MNs (Cullheim and Kellerth 1981; Eccles et al. 1954; Renshaw 1946; Schneider and Fyffe 1992), for the most part in the same or adjacent segments (Jankowska and Smith 1973; Kirkwood et al. 1981; Ryall et al. 1971; Saywell et al. 2013; van Keulen 1979) (Fig. 1*B*, *h*). In addition to projecting to the MNs that excite them (Moore et al. 2015), Renshaw cells project to homonymous and synergistic MNs (Eccles et al. 1954, 1961a; Renshaw 1946), and, as with other systems that coactivate α- and γ-MNs, Renshaw cells inhibit γ-MNs too (Ellaway 1971), although likely to a lesser extent than they inhibit α-MNs (Ellaway and Murphy 1981; Granit et al. 1957) (Fig. 1*B*, *i*).

It is not clear whether the effects of Renshaw inhibition differ on different MN types. Initially, it was thought that Renshaw inhibition was weighted toward tonically active MNs (i.e., S > FR > FF) (Friedman et al. 1981; Granit et al. 1957). On the other hand, synaptic currents produced by Renshaw cells have been shown to be similar across different MN types (Lindsay and Binder 1991). Whether there are differential functional effects of this inhibition is not known. For example, persistent firing in type S MNs (Lee and Heckman 1998) may be particularly sensitive to Renshaw inhibition (Bui et al. 2008; Hultborn et al. 2003). The specific effects of Renshaw inhibition of γ-MNs are not known.

The functional role of Renshaw inhibition is not clear, with many hypotheses having been raised (Alvarez and Fyffe 2007; Brownstone et al. 2015; Hultborn et al. 1979; Windhorst 1996). For example, Renshaw inhibition may serve to limit MN firing (Noga et al. 1987), curtail plateau potentials and persistent firing (Bui et al. 2008; Hultborn et al. 2003), or even support high firing rates of MNs (Obeidat et al. 2014). In addition, it has been proposed that they serve at the motor pool level to restructure recruitment (Hultborn et al. 1979) or at the microcircuit level to guide the tuning of MN properties (Brownstone et al. 2015).

Examination of the evolution and distribution of Renshaw cells has failed to unearth a unifying hypothesis of their function. Although about one-half of frog lumbar MNs have recurrent axon collaterals (Chmykhova and Babalian 1993), they do not lead to recurrent inhibition (Holemans and Meij 1968). There is evidence of a Renshaw-like feedback pathway in lampreys (Quinlan and Buchanan 2008), and of Renshaw-type neurons in chicks (Wenner and O’Donovan 1999), showing that they arose early in vertebrate evolution. In mammals, Renshaw cells form circuits with MNs innervating intercostal (Kirkwood et al. 1981) and other MMC MNs (Jankowska and Odutola 1980), neck MNs (Brink and Suzuki 1987), and phrenic MNs (Lipski et al. 1985). In circuits for limb MNs, Renshaw cells are involved in proximal motor pools more so than distal-innervating pools: Renshaw cells appear to be absent in motor pools innervating intrinsic hand and foot muscles in humans, as well as in distal muscles of the cat fore and hind limbs (for review, see Illert and Kümmel 1999; Pierrot-Desseilligny and Burke 2005; Piotrkiewicz and Młoźniak 2016). They are also absent from other pools, such as those innervating muscles of mastication (Shigenaga et al. 1989; Türker et al. 2007). Hence, although Renshaw inhibition evolved during vertebrate evolution, these neurons are involved more so with the control of MNs innervating proximal large muscles than in those involved in control of digits.

Whereas it is clear that Renshaw cells are not involved in circuits for all vulnerable MNs, Renshaw cell circuits have not been identified in circuits of ALS-resistant motor pools. Motor axon collaterals have been observed from neurons in Onuf’s nucleus (Sasaki 1994), but Renshaw inhibition appears to be absent (Mackel 1979). There is no evidence of Renshaw-type inhibition of oculomotor neurons (Baker and Precht 1972; Sasaki 1963).

In summary, Renshaw cells are activated by axon collaterals of α-MNs, in particular those in proximal motor pools that are vulnerable in ALS, and in turn inhibit α- and γ-MNs in homonymous and synergist MN pools.

## HYPOTHESIS: MICROCIRCUIT IMBALANCE RESULTING FROM MOTONEURON DEATH LEADS TO DISEASE PROGRESSION

**Fig. 2.**
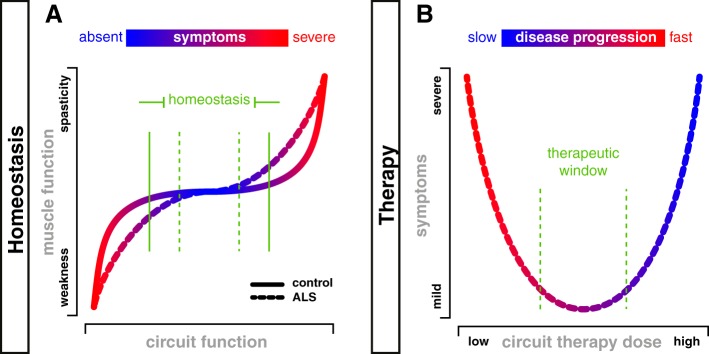
Homeostasis and microcircuit therapy: targeting microcircuits to slow progression? *A*: chair-shaped homeostatic curve (Nijhout et al. 2014) demonstrating a region in which normal motor function can be maintained despite increases and decreases in circuit function (solid green vertical bars). Increases beyond this range would lead to positive motor symptoms such as spasticity, whereas reductions would lead to weakness. As circuits degenerate in amyotrophic lateral sclerosis (ALS) and fewer α-motoneurons (α-MNs) are available to these circuits, the homeostatic plateau would narrow (dashed green vertical bars). *B*: microcircuit therapy for ALS, as defined here, would be aimed at reversing at least one of the arrows in Fig. 1. For example, a therapy to reduce γ-MN activity, or to increase Renshaw cell activity, could reverse the imbalance in these circuits, potentially slowing MN death as depicted by the color scale. We predict that this would reduce symptoms by preserving α-MNs. However, at higher “doses,” such therapy could in itself lead to weakness through reducing α-MN activity. We suggest that there would be a therapeutic window (dashed green vertical bars) in which progression could be slowed and the duration of time that people will have functional muscle contraction would increase, thus leading to improvements in quality of life.

In ALS, the underlying neurodegenerative process, regardless of how it starts, ultimately leads to death of α-MNs. FF MNs degenerate first, followed by FR MNs, whereas S MNs are well preserved until late stages of the disease (Frey et al. 2000; Pun et al. 2006). Clearly, homeostatic mechanisms compensate for this loss given that the disease is not symptomatic until at least 30% of a MN pool degenerates (Lalancette-Hebert et al. 2016; Zang et al. 2005) (Fig. 1, *C* and *D*, and Fig. 2*A*, solid line). These homeostatic processes (Nijhout et al. 2014) likely involve multiple sites in the nervous system, from descending to spinal cord circuits to MNs and to neuromuscular junctions (NMJs). Below we explore an hypothesis in which the imbalance of spinal cord microcircuits caused by the initial loss of FF MNs leads to runaway circuit function and hence progression of MN dysfunction and, ultimately, death. [We direct the reader to van Zundert et al. (2012) for a complementary discussion.]

The underlying assumption of this hypothesis is that MNs are susceptible to excitotoxic cell death (for review, see van Zundert et al. 2012; King et al. 2016). We do not review mechanisms of excitotoxicity, which have been well studied and reviewed elsewhere (e.g., Dong et al. 2009; King et al. 2016; Vucic et al. 2014). Briefly, excessive activation of glutamate receptors can lead to neuronal death via the resulting high levels of intracellular calcium activity. This process has been known for decades and used experimentally to produce excitotoxic lesions via focal injections of kainic acid, a glutamate receptor agonist (e.g., Coyle and Schwarcz 1976; McGeer and McGeer 1976). Pathologically, excitotoxicity has been well studied, for example, in stroke, in which death of cells at the stroke core results in a high concentration of extracellular glutamate, which subsequently results in further neuronal death (for review, see Arundine and Tymianski 2004). Thus it is well established that excessive glutamate receptor activity can lead to neuronal death.

The hypothesis we present is also based on two key underlying principles: *1*) γ-MNs are not affected in ALS (Mohajeri et al. 1998; Lalancette-Hebert et al. 2016; Vaughan et al. 2015); and *2*) during most movement, α-MNs and γ-MNs are for the most part coactivated (vide supra; Granit 1975; Grillner 1969; Hagbarth 1993; Sjöström and Zangger 1975, 1976).

We will assume that the first step in the degenerative process is α-MN dysfunction (Fig. 1, asterisks), defined as a reduction in the capacity of a MN to activate its associated muscle fibers appropriately for the task at hand. α-MNs are known to be affected at presymptomatic stages (Schütz 2005), so the homeostatic processes would start very early, long before symptoms appear (Fig. 1, *C* and *D*). For a given input to a motor pool, the associated loss of NMJ activation, particularly affecting fast, high-force-producing muscle fibers, would result in a reduction in the force of muscle contraction. The homeostatic responses to this reduction would be aimed at facilitating force production such that normal movement could proceed. These responses could include the following: *1*) changes at NMJs, including changes in synaptic transmission at the NMJ (Tremblay et al. 2017) and/or collateral sprouting at the NMJ following retraction of axon terminals such that slow motor axons transiently reoccupy vacated fast NMJs (Hegedus et al. 2008; Pun et al. 2006; Schaefer et al. 2005); *2*) changes in MN physiology, including increased excitability or increased glutamate receptors to produce higher rates of firing in response to a given input (although it is unlikely that these intrinsic changes contribute to MN death) (Leroy et al. 2014); and/or *3*) changes in circuits, including increased synaptic input to drive MNs to higher firing rates.

Given that initial dysfunction lies in the high force-producing activity of FF MNs, it is likely that homeostatic responses at the NMJ alone would be insufficient to compensate for this loss, and the homeostatic response of the nervous system would be to increase the firing rates of MNs. Although this could be accomplished in a cell autonomous process, e.g., by increasing membrane voltage-gated calcium channels (Heckman et al. 2003) or increasing available glutamate receptors, it seems likely that cell autonomous processes have evolved to maintain firing rate within set limits for any neuron type (O’Leary et al. 2014; Turrigiano and Nelson 2004). That is, in the absence of a Hebbian process, it seems unlikely that MNs would autonomously increase their firing frequency beyond their normal operating range. We thus propose that a key driving homeostatic response lies in circuit function, that is, increasing excitation of MNs by premotor circuits (Jiang et al. 2009) (Fig. 1, *C* and *E*, *1*).

This increased activity in premotor circuits would lead to increased output of functional α-MNs as well as γ-MNs, with the former being the homeostatic response leading to restoration of force production. As an increasing number of MNs becomes affected, the homeostatic range would narrow, because it would become increasingly difficult for the remaining functional MNs and their associated circuits to produce the required force output (Fig. 2*A*, dashed line). In addition, this increased input to the motor pool would lead to increased γ-MN activity, as well (Fig. 1, *C* and *E*, *3*), such that intrafusal fibers would contract out of proportion to extrafusal fibers (Fig. 1, *C* and *E*, *2* and *4*). Increased attention to the task secondary to any perceived weakness may also increase γ-MN activity (Hospod et al. 2007). This in turn would lead to a relative increase in spindle sensitivity and thus increased afferent activity from the spindles, perhaps even during muscle contractions (Fig. 1*C*, *5*). This could initially assist the homeostatic response, compensating for the reduction in motor pool output via “servo-assistance” (Hagbarth 1993; Watanabe and Hirayama 1976). Furthermore, such a compensatory response would be weighted to α-MNs, leading to some normalization of α-γ balance (although weighted to S over F). However, both the increased premotor input and the spindle afferent input will lead to increased activation of glutamate receptors (Fig. 1*C*, *6*) and will thus contribute to excitotoxicity (Fig. 1, Glu). That is, following initial compensatory processes, there would be an escape from the homeostatic responses (Nijhout et al. 2014), ultimately resulting in MN degeneration and weakness (to the left on the curve in Fig. 2*A*).

Interestingly, a significant proportion of humans with ALS have abnormal sensory function, as well (Dyck et al. 1975; Hammad et al. 2007). Recent evidence from two different animal models has shown that the peripheral innervation of spindles by group Ia and II fibers is diminished in the presymptomatic stages of disease, even though the sensory neuron somata are unaffected at this time point (Dal Canto and Gurney 1995; Vaughan et al. 2015), and central synapses are affected only late in the disease process (Vaughan et al. 2015). These changes occur in parallel with α-MN degeneration in one animal model of familial ALS (SOD1^G93A^) but before changes in motor axons in another (TDP43^A315T^) (Vaughan et al. 2015). This reduction in peripheral innervation would limit the excitotoxic effects described above (Fig. 1*E*, thinner red line compared with Fig. 1*C*), whether the degeneration is a primary effect of the disease or a homeostatic response to an α-γ mismatch.

However, this peripheral circuit would not be the only dysfunctional MN microcircuit. In motor pools that have Renshaw inhibition, the reduction in α-MN activity would also lead to a loss of input to Renshaw cells (Fig. 1*F*, *7*), which would reduce Renshaw pool activity. Although this would diminish α-MN inhibition (Fig. 1*F*, *8*) and thus could aid in a homeostatic compensatory process leading toward normalization of muscle contraction, it would also diminish γ-MN inhibition (Fig. 1*F*, *9*). That is, the loss of Renshaw cell activation by α-MNs would lead to a further imbalance of α-γ output (Fig. 1, *C* and *D*, *2* and *4*), leading to a relative increase in spindle contraction (Fig. 1*C*, *5*). This in turn would lead to an increase in spindle afferent activity (Fig. 1*C*, *6*) and, as above, contribute to excitotoxic cell death (Fig. 1, Glu). In addition, given that Renshaw cells have been shown to limit plateau potentials in α-MNs (Bui et al. 2008; Hultborn et al. 2003), reduction in their activity could also lead to hyperexcitability of α-MNs and increased calcium entry, thus directly contributing to excitotoxic cell death. Although we propose that this circuit dysfunction may contribute to the degenerative process, it is clear that it is not necessary for MN degeneration, because not all vulnerable MN pools have Renshaw cell circuits.

Note that the selective vulnerability of large fast MNs/motor units to ALS may initially be due to their specific cellular profile, that is, the combination of particular molecular, physiological, and metabolic properties. However, the central and peripheral pattern of connectivity of these MNs, that is, their local microcircuits, may play a key role in the progression and propagation of MN degeneration.

Is there evidence of reduced Renshaw cell activity in ALS? The role of Renshaw cells in ALS has been reviewed elsewhere (Mazzocchio and Rossi 2010; Ramírez-Jarquín et al. 2014). There is evidence that recurrent inhibition is reduced in people with ALS (Raynor and Shefner 1994). At early asymptomatic stages in ALS animal models, Renshaw cells are spared (Knirsch et al. 2001; Morrison et al. 1996) (Fig. 1*D*, *8* and *9*). At these presymptomatic stages, there is evidence of axonal sprouting of Renshaw cells leading to transient upregulation of glycinergic synapses on MNs (Chang and Martin 2009; Wootz et al. 2013). However, as the disease progresses, Renshaw cells receive progressively less input from MNs, with some Renshaw cells being completely denervated (Wootz et al. 2013). A proportion of Renshaw cells then dies over the course of the disease (Chang and Martin 2009). Thus there is evidence that a reduction in MN inputs to Renshaw cells leads to a reduction in recurrent inhibition but that Renshaw cells initially compensate by sprouting on remaining viable MNs (Wootz et al. 2013). These initial changes would be homeostatic, but as disease progresses, there would be an escape from this homeostatic process.

Although there is no evidence to date of α-γ imbalance in ALS, the hypothesis could be readily tested. Though studies of short-latency reflexes in reduced preparations in ALS models can highlight changes in the integrity of these pathways (the afferents, the synapses on MNs, and the motor output), they reveal neither afferent nor γ-MN activity in the intact animal (Jiang et al. 2009). Reflex studies in the awake, intact animal could examine underlying spindle tone compared with that in wild-type mice, but perhaps the ideal experiment would involve microneurography in humans at different stages of ALS, where activity in identified axons can be recorded (Dimitriou 2014; Edin and Vallbo 1990). The hypothesis presented here could thus be readily studied.

One clinical sign that would be seen with this maladaptive plasticity would be spasticity. ALS patients with supraspinal disease tend to have a greater degree of spasticity than those with predominantly spinal disease (Gordon et al. 2009). The mechanisms underlying this spasticity may therefore be similar to those in spinal cord injury, such as changes in serotonin receptors (Murray et al. 2010) or chloride reversal potentials (Boulenguez et al. 2010). However, the α-γ MN imbalance described above could also contribute to spasticity, with muscle stretches producing excessive spindle afferent activity due to the high γ-MN tone (Gladden et al. 1998). That is, if sufficient numbers of α-MNs remain functional, the relative increase in γ-MN activity would lead to an escape to the right of the homeostatic curve (Fig. 2*A*).

In summary, we propose that the homeostatic response to reduced neuromuscular activity following early MN dysfunction would be for the nervous system to increase inputs to α-MNs. With ongoing loss of α-MN function, these increased inputs would lead to a shift in the α-γ balance, leading to increased afferent input to α-MNs. Concomitantly, in some motor pools there would be a reduction in Renshaw cell inhibition resulting from reduced MN excitation of Renshaw cells. The changes to both Ia-MN and MN-Renshaw cell circuits would initially be homeostatic but would ultimately lead to excitotoxic death of MNs. Furthermore, we note that the increased inputs to MNs will affect more than a single motor pool [consider muscle synergies, e.g., Takei et al. (2017); respiratory-locomotor coupling, e.g., Romaniuk et al. (1994); reticulospinal effects on multiple body segments, e.g., Drew and Rossignol (1990)] and hence could contribute to symptomatic spread of the disease from one MN pool to another (Ravits 2014). That is, though not a cause of ALS, such circuit dysfunction could contribute to its progression within and beyond motor pools.

## TARGETING MICROCIRCUITS TO SLOW DISEASE PROGRESSION?

If this hypothesis is correct, then therapies that target this microcircuit dysfunction should slow the progression of disease (Fig. 2*B*). Microcircuit therapy for ALS, as defined in this article, could thus be devised to target, for example, γ-MNs, to reduce their activity, or Renshaw cells, to increase their activity. Whereas we predict on the basis of the hypothesis presented here that either approach would slow the progression of the disease, overzealous treatment of either could also lead to a reduction in MN output and hence weakness. We suggest, however, that there would be a therapeutic window in which disease progression could be slowed and quality of life thus improved by increasing the duration of time that people will have functional muscle contraction (Fig. 2*B*).

There is no evidence in humans that such therapies might be effective, and we do not yet have the knowledge or methods to specifically target these microcircuits, but there is some recent evidence provided by animal models of ALS. In two models, the progression of disease was slowed concomitant with a reduction in γ-MN activity. In the first, muscle spindles were targeted, and their degeneration was associated with a loss of γ-MNs (Lalancette-Hebert et al. 2016). In the second, γ-MNs were selectively targeted genetically, reducing their population by half. In both instances, there was a slower rate of α-MN loss, signs of ALS were significantly delayed, and survival was prolonged (Lalancette-Hebert et al. 2016). These findings are consistent with the hypothesis presented here.

To target these circuits in humans, if there are sufficient data to support the concept, a gene therapy approach could be considered (e.g., https://clinicaltrials.gov/ct2/show/NCT03306277 for spinal muscular atrophy). As we learn more about gene expression profiles in mature MNs or Renshaw cells, for example, promoters for these genes could be used in viral approaches to drive expression confined to these populations. For example, introduction of potassium channels to γ-MNs or inhibitory “designer receptors exclusively activated by designer drugs” (DREADDs) could be expressed to reduce activity (in the latter case, with oral clozapine). Perhaps the advantage of a DREADD approach would be that dosage could be titrated and the therapeutic window thus defined (Fig. 2*B*).

In neurodegenerative diseases, much research is necessarily focused on causation, with the concept being that identification of the cause of the disease will lead to strategies to prevent or cure the disease. On the other hand, clinical treatment is focused on symptom amelioration, because that is where our state of knowledge is. We suggest that there is a third possibility for treatment that may be unrelated to the cause of the disease: treatment of dysfunctional microcircuits to slow the progression of the disease.

## GRANTS

R. Brownstone is supported by Brain Research UK, and the motoneuron work in his laboratory is supported by Wellcome (grant no. 110193).

## DISCLOSURES

No conflicts of interest, financial or otherwise, are declared by the authors.

## AUTHOR CONTRIBUTIONS

R.M.B. conceived and designed research; C.L. prepared figures; R.M.B. and C.L. drafted manuscript; R.M.B. and C.L. edited and revised manuscript; R.M.B. and C.L. approved final version of manuscript.
